# Evaluations of diffusion tensor image registration based on fiber tractography

**DOI:** 10.1186/s12938-016-0299-2

**Published:** 2017-01-10

**Authors:** Yi Wang, Yu Shen, Dongyang Liu, Guoqin Li, Zhe Guo, Yangyu Fan, Yilong Niu

**Affiliations:** 1 School of Electronics and Information, Northwestern Polytechnical University, Xi’an, 710072 China; 2 School of Marine Science and Technology, Northwestern Polytechnical University, Xi’an, 710072 China

**Keywords:** DTI, Registration algorithms, Evaluation, Tractography

## Abstract

**Background:**

Diffusion Tensor Magnetic Resonance Imaging (DT-MRI, also known as DTI) measures the diffusion properties of water molecules in tissues and to date is one of the main techniques that can effectively study the microstructures of the brain in vivo. Presently, evaluation of DTI registration techniques is still in an initial stage of development.

**Methods and results:**

In this paper, six well-known open source DTI registration algorithms: Elastic, Rigid, Affine, DTI-TK, FSL and SyN were applied on 11 subjects from an open-access dataset, among which one was randomly chosen as the template. Eight different fiber bundles of 10 subjects and the template were obtained by drawing regions of interest (ROIs) around various structures using deterministic streamline tractography. The performances of the registration algorithms were evaluated by computing the distances and intersection angles between fiber tracts, as well as the fractional anisotropy (FA) profiles along the fiber tracts. Also, the mean squared error (MSE) and the residual MSE (RMSE) of fibers originating from the registered subjects and the template were calculated to assess the registration algorithm. Twenty-seven different fiber bundles of the 10 subjects and template were obtained by drawing ROIs around various structures using probabilistic tractography. The performances of registration algorithms on this second tractography method were evaluated by computing the spatial correlation similarity of the fibers between subjects as well as between each subject and the template.

**Conclusion:**

All experimental results indicated that DTI-TK performed the best under the study conditions, and SyN ranked just behind it.

## Background

Diffusion Tensor Magnetic Resonance Imaging (DT-MRI, also known as DTI) [1] is a magnetic resonance imaging technique. DTI measures the diffusion properties of water molecules in tissue and creates images showing physiological information such as neural bundles, which cannot be obtained by other imaging methods. DTI can be used to infer some microscopic features and organizational information regarding the structural anatomy of tissues, especially the orientation of fibrous tissues, which has been used extensively to study white matter fiber tracts [2–4]. DTI plays an important role in the in vivo study of anatomical structures and functional connectivity throughout the brain.

Many DTI registration algorithms have been proposed. With respect to data processing, registration methods can be divided into three categories: scalar image-based registration algorithms, tensor image-based registration algorithms, and fiber bundle-based registration algorithms. Scalar image-based registration algorithms use scalar images derived from DTI images, which are mainly fractional anisotropy (FA) images, to perform registrations [2]. Voxels in tensor images are displaced according to the displacement field obtained with scalar registration and then tensor reorientation is performed. Different from scalar image-based registration algorithms, tensor image-based registration algorithms use the tensor instead of the scalar to perform registrations [5]. Meanwhile, fiber bundle-based registrations use fiber bundles tracked from the DTI images to perform registration directly [6, 7], but it spends more time on fiber tractography from DTI images according to regions of interest (ROIs).

Currently, there is no standard method for evaluating the performance of DTI registrations. As such, it is necessary to develop evaluation strategies on the topic. However, development of DTI registration evaluation strategies is challenging because each DTI registration algorithm has advantages and disadvantages for different ROIs, and a single evaluation strategy cannot be broadly applied to all algorithms.

Previous studies have utilized evaluation criteria based on regional matching. In 2000, Basser et al. [3] proposed the use of two diffusion tensor eigenvalues-eigenvectors overlapping rates (Overlap of Eigenvalue–eigenvectors Pairs). In 2002, Jones et al. [4] proposed the use of a tensor-normalized standard deviation (Normalized Standard Deviation of Tensors) and Dyadic Coherence to assess matching performance. Both evaluation criteria take advantage of the direction of the diffusion anisotropy value and principal eigenvector. In 2006, Zhang et al. [5] used the tensor Euclidean distance (Euclidean Distance) and the tensor deviation Euclidean distance (Euclidean Distance of the Deviatoric Tensor) to evaluate the spatial normalization accuracy. In 2007, Van Hecke et al. [8] proposed using the angles of diffusion tensor eigenvalues-eigenvectors as evaluation criteria. However, the most direct way to evaluate the performance of registration algorithms is with a similarity metric of tensor. In 2007, Klein [9, 10] proposed the use of voxels and surface overlaying rate (Volume and Surface Overlap), and registration accuracy was assessed by computing the overlap of segmented edges. Precision and convergence properties were studied by comparing deformation fields. In 2011, Wang et al. [2] proposed a partial area matching quality criterion (Regional Matching Quality Criterion). In 2012, Adluru et al. [11] used the Euclidean distance, Euclidean norm, cross-correlation, and eigenvalue-eigenvector pair of overlapping rate assessment criteria. In 2013, de Groot et al. [12] used the spatial similarity metric as the assessment criteria.

Currently, other scholars are studying evaluation criteria based on fiber bundles. However, this technique requires that the fiber information be extracted prior to evaluation. Tract extraction techniques are mostly semi-automatic, although small or thin fiber tracts are difficult to track and extract, so application of this technique is relatively limited. In 2006, Zhang et al. [5] calculated the average distance of points in two corresponding tracts as an evaluation parameter. In 2007, Mayer et al. [6] calculated the mean squared error (MSE) between model and target fibers before and after image registration to validate their registration algorithm. In 2010, Shadmi et al. [7] calculated the MSE and the residual MSE (RMSE) between the warped model and the target fiber sets to assess their registration algorithm. In 2011, Wang et al. [2] proposed a fiber property profile approach to perform evaluation. In 2013, de Groot et al. [12] proposed the fiber-based spatial similarity metric to assess the registration algorithms.

However, there are some problems with the existing evaluation techniques. In 2009, Klein et al. [13] evaluated performances of registrations for anatomic regions and the whole voxels of brain using the overlap rates on voxels and surfaces, the similarity of voxels and measuring distances. They evaluated 14 registration algorithms, but compared the scalar image-based registration algorithms without tensor-based registration algorithms. In 2011, Wang et al. [2] evaluated eight registration algorithms, including registration algorithms based on scalar images and tensor images. However, the Wang et al. study only used two evaluation criteria on infantile data which had lower FA value and signal-to-noise ratio compared to adult datasets. Since results differ between registration of infantile and adult images using the same technique, adult data was selected for this study and is easily accessed in several open sources. In 2013, de Groot et al. [12] proposed use of the spatial similarity metric based on the fibers accessed through the registration algorithms, however only two algorithms were compared.

The performance metrics based on similarity of tractography are independent of any particular similarity matrix derived from scalar or higher order images, and are adopted in most registration approaches. It should also be noted that optimal white matter tract alignment is most closely linked to the eventual registration goal of obtaining anatomical correspondence in white matter [8]. In this study, the data from healthy individuals was used to evaluate the DTI registration algorithm based on white matter fiber tracts.

Six well-known open source DTI registration algorithms (Elastic, Rigid, Affine, DTI-TK, FSL and SyN) were investigated. The performance of each registration algorithm was evaluated by computing the distances and intersection angles between fiber tracts, as well as with the FA profiles along the fiber tracts using deterministic streamline tractography. Also, the mean squared error (MSE) and the residual MSE (RMSE) of fibers originating from registered subjects and the template were calculated to assess the registration algorithm. The performance of each registration algorithm was also evaluated by computing the spatial correlation similarity of the fibers between the subjects as well as between each subject and the template using probabilistic tractography.

## Methods

### Materials

Diffusion MRI Data: The open-access IXI dataset from the Hammersmith Hospital of London was used (http://www.brain-development.org). A 3 Tesla Philips MRI scanner was used to scan the healthy subjects. The spatial resolution of the images was 1.7409 × 1.7355 × 1.9806 mm, resulting in volume data for the head of 128 × 128 × 64 voxels. Diffusion-weighted images were acquired along 15 unique gradient directions with *b* = 1000 s/mm^2^ (repetition time = 11,894.44 ms; echo time = 51 ms). Additional imaging parameters can be found at the image library website.

#### Subject and template

In this paper, 10 subjects were chosen at random from the dataset (mean age = 51.549 years, min age = 30.89 years, max age = 74.01 years, including: 5 males, mean age = 51.586 years, min age = 30.89 years, max age = 63.68 years; and 5 females, mean age = 51.512 years, min age = 33.76 years, max = 74.01 years). For the template, although DTI-TK (http://www.nitrc.org/projects/dtitk/) could produce a good template with sufficient DTI information to perform tractography, using DTI-TK would bias the analysis since it is compared here. So another subject with quality inspection was chosen from the same dataset at random to serve as the template (male, age = 37.83 years).

### Pre-processing

The Brain Extraction Tool (BET) within the FMRIB software Library (FSL) was used to extract brain tissue for each subject and template. The mask used for skull stripping was generated from each subject or template individually and checked manually. Before tensor estimation, diffusion-weighted images (DWIs) from 15 diffusion gradient directions were eddy-current corrected with eddy tool in FSL, which is a tool to correct eddy current-induced distortions and subject movements in diffusion data [14].

### Registration methods

In accordance with the work of Wang et al. [2], we chose six relatively mature open source registration algorithms to evaluate. All of the subjects were normalized at first. The six DTI registration algorithms investigated in this paper are described in detail below.

In 2000, Alexander et al. [15] applied Elastic Registration Algorithm (referred to as Elastic in this paper) to diffusion tensor image. It can be performed with Advanced Normalization Tools (ANTs) (http://www.nitrc.org/projects/ants). In 1999, Studholme et al. [16] proposed Rigid body registration algorithm (referred to as Rigid in this paper). It also can be performed with ANTs, and it is one of the simplest algorithms of image registration.

In 2005, Leemans et al. [17] rendered an algorithm based on multi-channel affine registration, and the mutual information was used for similarity criteria (referred to as Affine in this paper). It is often performed before most deformation registrations and available through ANTs.

In 2006, Zhang et al. [5] developed a diffeomorphic deformable tensor registration technique (termed DTI-TK) (http://www.nitrc.org/projects/dtitk/). It is the only open source and nonlinear tensor-based registration algorithm (referred to as DTI-TK in this paper).

In 2008, Andersson et al. [18] developed a B-spline registration algorithm based on the sum-of-squared differences performed by FSL (http://www.nitrc.org/projects/fsl) (referred to as FSL in this paper).

In 2008, Avants et al. [19] developed a symmetric image normalization method based on mutual correlation (referred to as SyN in this paper) again with ANTs.

**Fig. 1:**
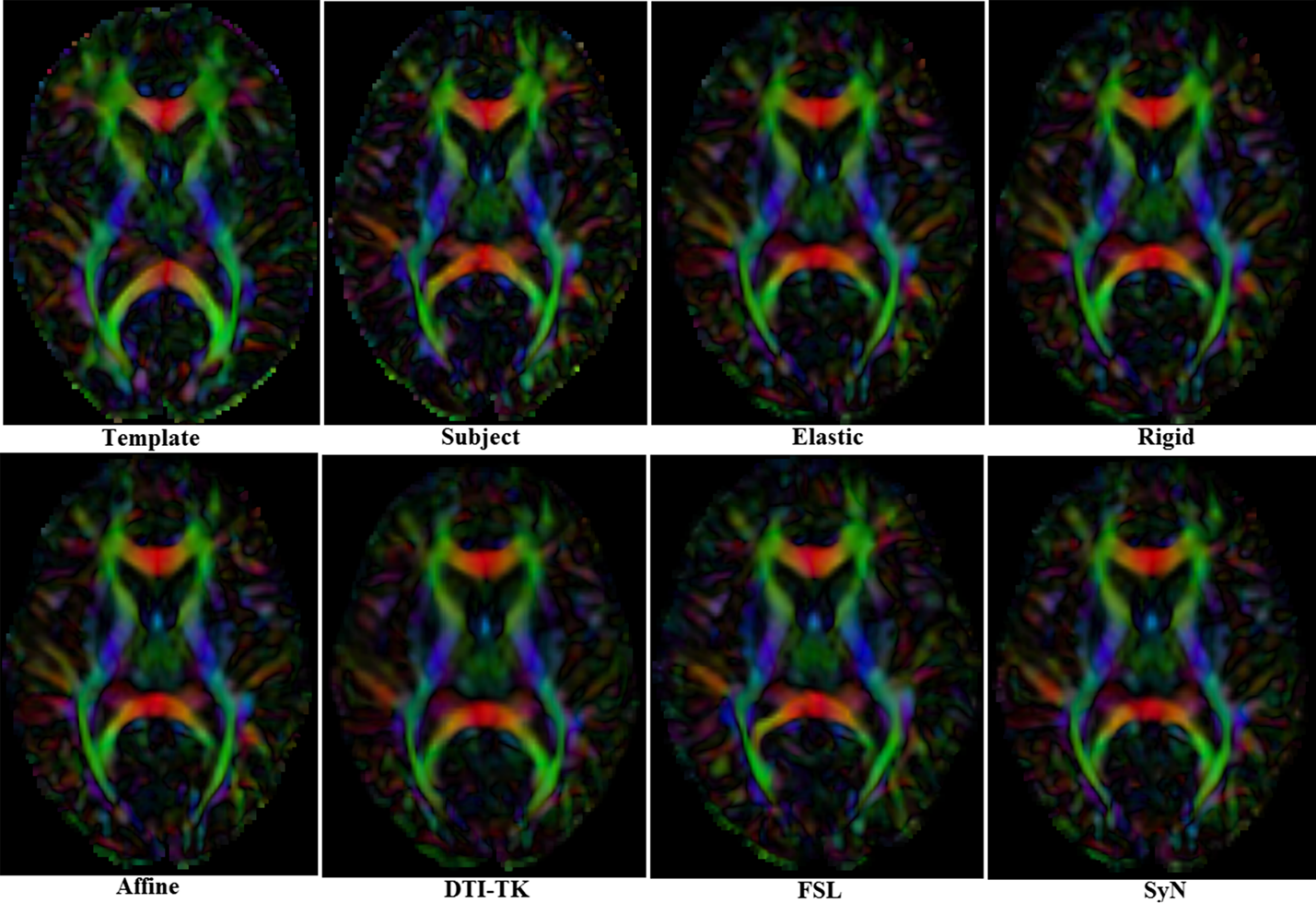
Template, a subject and the results of 6 different registration algorithms

The registration algorithms discussed above were mainly applied using FA scalars except DTI-TK. For FA-based registrations, the tensor reorientation was completed through the preservation of principal directions (PPD) [8]. The results of each registration algorithm are shown in Fig. 1.

### Evaluation methods

In this paper, deterministic streamline tractography [20, 21, 22] and probabilistic tractography [12, 23, 24, 25] were used to track fibers separately. Deterministic streamline tractography is used to evaluate the DTI registration based on the distances and intersection angles between fiber tracts as well as the fiber property profiles, MSE, and RMSE. Probabilistic tractography is used to evaluate DTI registration based on the spatial similarity metric.

**Fig. 2:**
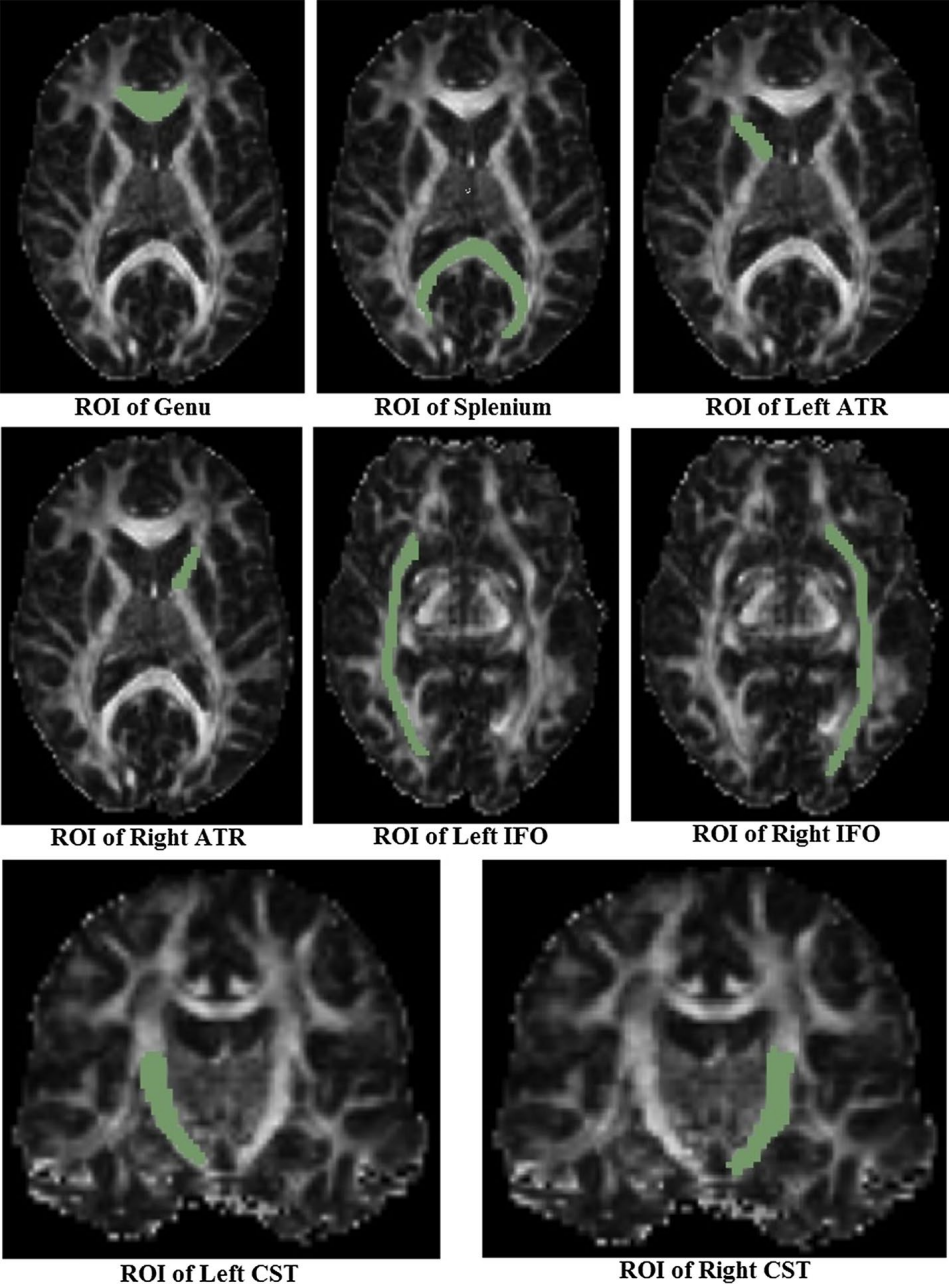
ROIs on template’s FA image

**Fig. 3:**
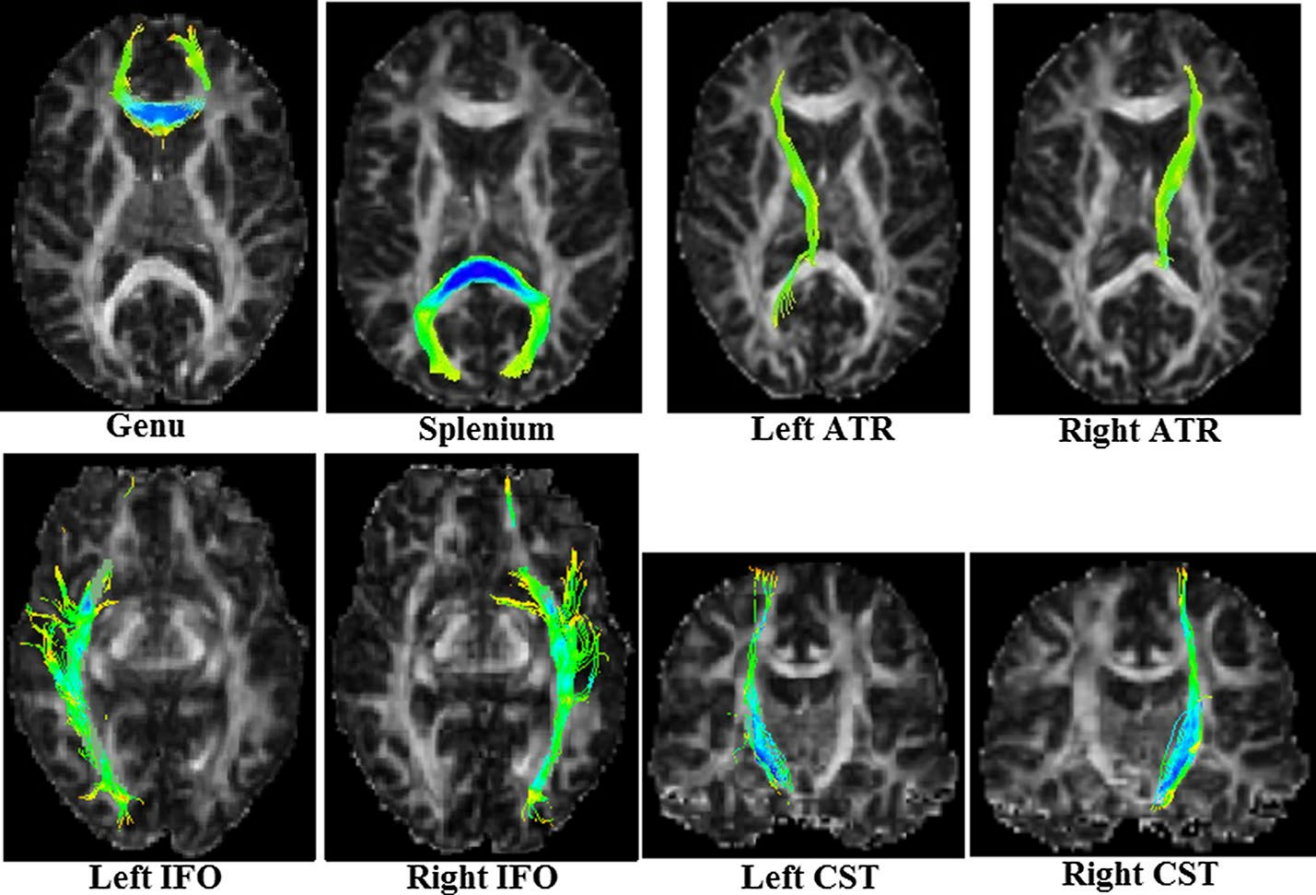
Fibers of the template

To perform deterministic streamline tractography with FACT (Fiber Assessment by Continuous Tracking) [21, 22], eight different ROIs [2, 26–29] were manually drawn on FA maps according to the work of Zhang et al. [5]. The corresponding fiber tracts of interest to this study are: the knee of the Corpus Callosum (Genu of the corpus callosum, namely Genu), the splenium of the Corpus Callosum (the Splenium of the corpus callosum, namely Splenium), the left and right Thalamic radiations (Anterior Thalamic Radiations, namely ATR), the left and right fronto-occipital fasciculus (Inferior Fronto-occipital Fasciculi, namely IFO) and the left and right cortical/corticospinal tracts of the medulla oblongata (Corticospinal/Corticobulbar tracts, namely CST). In evaluation methods based on distance between fiber tracts, as well as MSE and RMSE of fibers, the fibers of each subject and the template are tracked individually with the same ROIs drawn on the template FA image [2, 9, 11, 26]. In evaluation methods based on the FA profiles along the fiber tracts and intersection angles between fiber bundles, fibers of the template are tracked first, and then fibers of each subject were obtained by directly mapping the template fibers onto the same positions [2]. ROIs on the template FA image are shown in Fig. 2 and the fibers of eight ROIs on the template are shown in Fig. 3.

**Fig. 4:**
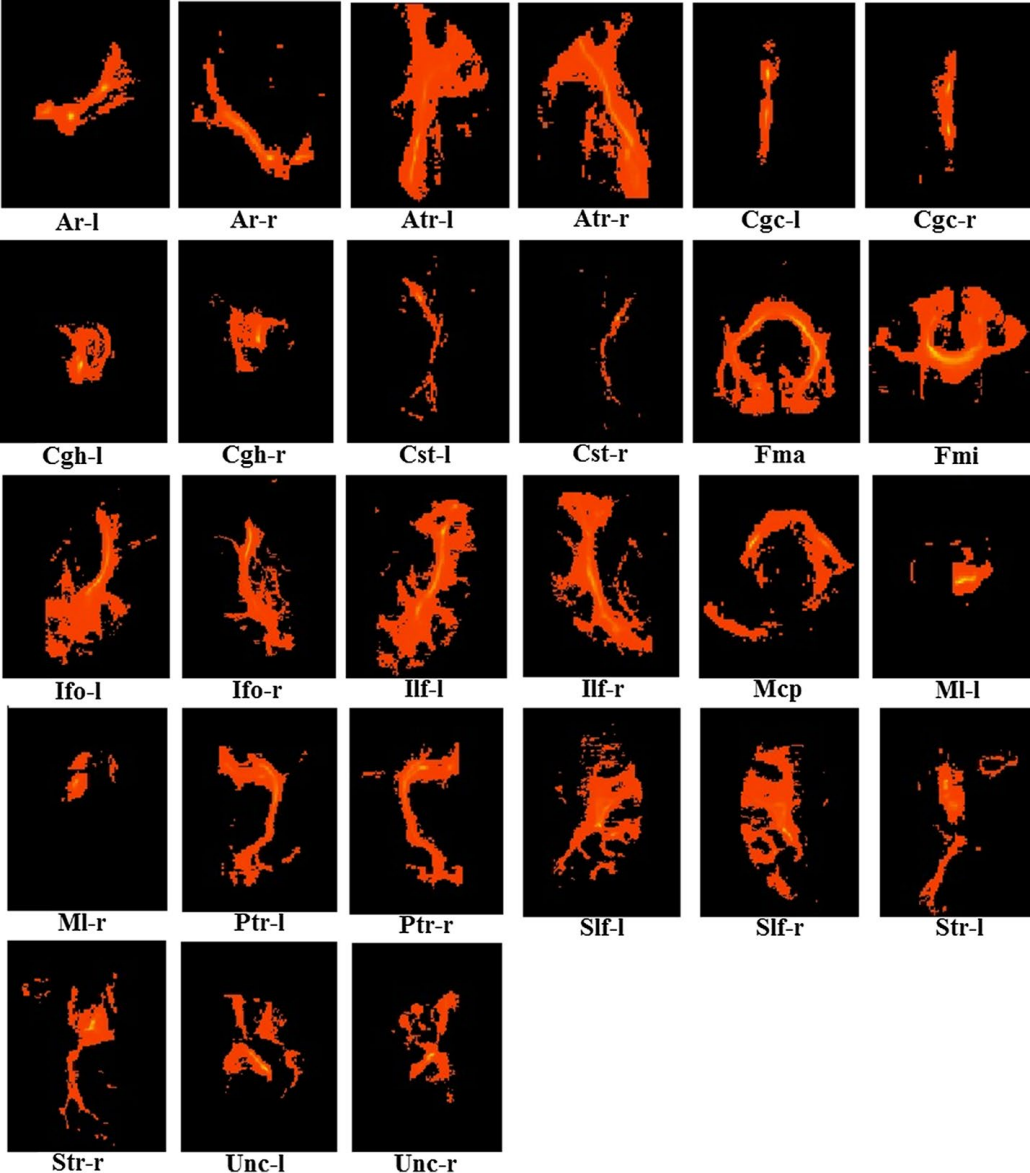
Results of probabilistic tractography on the template

Probabilistic tractography was performed with PROBTRACKX [10, 24, 25] available in FSL. The masks used were based on the protocols described by Mori [26, 27], Stieltjes et al. [28], and Wakana et al. [30, 31], and 27 different ROIs of DTI were used to track fiber bundles. The various ROIs are shown in Table 1 and the results of probabilistic tractography are shown in Fig. 4. All of the tracking parameters were set to the default values available in FSL [10].

**Table 1:** The seeds used in this paper, tracts with left/right homologues are listed [23, 26, 29, 24, 31]

ROI	Left/right
Acoustic radiation (Ar)	+
Anterior thalamic radiation (Atr)	+
Superior thalamic radiation (Str)	+
Posterior thalamic radiation (Ptr)	+
Superior longitudinal fasciculus (Slf)	+
Inferior longitudinal fasciculus (Ilf)	+
Inferior fronto-occipital fasciculus (Ifo)	+
Uncinate fasciculus (Unc)	+
Cingulate gyrus part of cingulum (Cgc)	+
Parahippocampal part of cingulum (Cgh)	+
Forceps minor (Fmi)	−
Forceps major (Fma)	−
Middle cerebellar peduncle (Mcp)	−
Medial lemniscus (Ml)	+
Corticospinal tract (Cst)	+

‘+’ shows that the ROI is different across the left and the right

#### Evaluation method based on distance between fiber tracts

In most cases, the objective function of registration is the registration for anatomical structures. So performances of registration assessment also should be the measurement of anatomical structures.

In 2006, distances between fiber tracts were proposed by Zhang et al. [5] to evaluate registration performance:

1 $$f = \frac{1}{{{\text{F}} + {\text{G}}}}\left( {\sum\limits_{{F_{i} \in F}} {\mathop {\hbox{min} }\limits_{{G_{j} \in G}} {\text{d}}\left( {F_{i} ,G_{j} } \right) + \sum\limits_{{G_{j} \in F}} {\mathop {\hbox{min} }\limits_{{F_{i} \in G}} {\text{d}}\left( {F_{i} ,G_{j} } \right)} } } \right)$$

where *f* is the Hausdorff distance, d is a pairwise distance between two fibers, F and G are two fiber bundles, $\mathop {\hbox{min} }\limits_{{G_{j} \in {\text{G}}}} {\text{d}}\left( {F_{i} ,G_{j} } \right)$ is the distance between the fiber $F_{i}$ and the fiber in G that is closest to $F_{i}$, and similarly, $\mathop {\hbox{min} }\limits_{{F_{i} \in {\text{G}}}} {\text{d}}\left( {F_{i} ,G_{j} } \right)$ is the distance between the fiber $G_{j}$ and the fiber in F that is closest to $G_{j}$. Equation (1) is symmetric with respect to the two fibers involved, and when two identical fiber bundles are perfectly aligned, it evaluates to zero. The lower the *f* value is, the better the registration performance is.

#### Evaluation method based on the MSE and RMSE of fibers

In 2007, Mayer et al. [6] calculated the MSE between model and target fibers before and after registration to verify the validity of their registration algorithm. In 2010, Shadmi et al. [7] calculated the MSE and the RMSE between a warped model and a target fiber sets to assess the registration algorithm.

2 $$MSE = \frac{1}{n}\sum\limits_{i = 1}^{n} {\left( {subject_{i} - template} \right)^{2} }$$

3 $$RMSE = \frac{{{\text{local}}\,{\text{MSE}}}}{{{\text{global}}\,{\text{MSE}}}}$$

In this study, $subject_{i}$ (*i* = 1, 2,…,*n*) represent registered fibers using different registration algorithms, and *template* is the corresponding fiber of the template. Table 2 shows the evaluation results of registration based on distances between fibers. Table 3 shows the MSE between each subject and template pair for each ROI. Table 4 shows the RMSE between each subject and template for each ROI.

**Table 2:** Evaluation results of registration based on distances between fibers

	Genu	Splenium	L-ATR	R-ATR	L-CST	R-CST	L-IFO	R-IFO	Mean
Elastic	0.5202	0.5509	0.4892	0.5264	0.4953	0.5005	0.4697	0.4533	0.5007
Rigid	0.5275	0.5097	0.4941	0.5693	0.5037	0.4960	0.4822	0.4704	0.5066
Affine	0.5346	0.5244	0.4984	0.5392	0.5000	0.4912	0.4664	0.4773	0.5039
DTI-TK	0.4876	0.4792	0.4872	0.4783	0.4736	0.4904	0.4549	0.4098	0.4701
FSL	0.4971	0.4860	0.4914	0.5023	0.4920	0.4656	0.4427	0.4053	0.4728
SyN	0.4835	0.4958	0.4788	0.5126	0.4748	0.4716	0.4548	0.4153	0.4734

**Table 3:** Evaluation results of registrations based on MSE of fibers

	Genu	Splenium	L-ATR	R-ATR	L-CST	R-CST	L-IFO	R-IFO	Mean
Elastic	0.0460	0.0505	0.0073	0.0099	0.0188	0.0307	0.0138	0.0088	0.0232
Rigid	0.0362	0.0844	0.0231	0.0174	0.0492	0.0659	0.0193	0.0161	0.0390
Affine	0.0805	0.0816	0.0119	0.0154	0.0264	0.0432	0.0230	0.0122	0.0368
DTI-TK	0.0033	0.0097	0.0033	0.0034	0.0056	0.0069	0.0054	0.0036	0.0052
FSL	0.0460	0.0517	0.0096	0.0127	0.0191	0.0285	0.0117	0.0073	0.0233
SyN	0.0046	0.0140	0.0044	0.0049	0.0061	0.0080	0.0059	0.0058	0.0067

#### Evaluation method based on the FA profiles along the fiber tracts

In 2011, Wang et al. [2] proposed a fiber property profile-based metric using normative correlation. Along each fiber bundle, FA profiles were calculated. For each registered subject, each fiber was recaptured with the same location as the fiber of the template. With the defined fiber bundles, FA curves of each fiber bundle were redefined, and then the corresponding mean FA curves were derived from the fiber bundles of the same ROI for all subjects.

#### Evaluation method based on intersection angles between fiber bundles

According to Wang’s paper [2], the fiber tracts from a template can be mapped to each registered subject to obtain the corresponding tracts with consistent positioning. That is, if a better registration is obtained, improved consistency in the anatomical structures will subsequently be achieved. Meanwhile, a better registration is indicative of a smaller intersection angle between the tracts and a subsequent increase in the corresponding cosine value.

4 $$\cos \alpha = \frac{{\sum\limits_{i} {\sum\limits_{j} {F_{i} \cdot G_{j} } } }}{{\left\| {\text{F}} \right\|\left\| {\text{G}} \right\|}}$$

Here, $F_{i}$ and $G_{j}$ are fibers of the template and one subject respectively, F and G are two fiber bundles, and the value of $\cos \alpha$ is between 0 and 1. The higher the value of $\cos \alpha$ is, the better the performance is. For each ROI, the final result represents an average value of $\cos \alpha$ across the fibers between all subjects and template.

#### Evaluation method based on spatial similarity between fiber tracts

The framework developed by de Groot et al. [12] was used to evaluate scalar or higher-order similarity matrices based on white matter tractography. With this method, the fiber tracts are obtained based on probabilistic tractography. A similarity matrix was used to assess the spatial correlation similarity matrix:

5 $$C = \frac{{\sum\nolimits_{i} {{\text{J}}_{i} {\text{K}}_{i} } }}{{\sqrt {\sum\nolimits_{i} {{\text{J}}_{i}^{2} } } \sqrt {\sum\nolimits_{i} {{\text{K}}_{i}^{2} } } }}$$

Equation (5) provides a measure of the voxel-wise similarity of the tracts density images (J and K) for two subjects. It computes over all voxels (*i*), and is bound on a 0–1 scale. A similarity matrix is calculated on the tract density images. A higher spatial correlation similarity indicates a better registration.

## Results

### Evaluation method based on distance between fiber tracts

Table 2 shows the average fiber distances between each subject and template pair of fibers where Genu, Splenium, L-ATR (left ATR), R-ATR (right ATR), L-CST (left CST), R-CST (right CST), L-IFO (left IFO), R-IFO (right IFO) are the eight fibers tracked by streamline fiber tracking algorithm of Deterministic Fiber Tractography for the template and subjects. “Mean” is the average value of each of the eight ROIs across all registration algorithms. For each ROI, the final result is the average distance of fibers between all subjects and template.

The average distances of each registration algorithm are presented in Table 2 DTI-TK had the lowest value and the SyN algorithm had the second lowest value. These results indicate that the DTI-TK registration algorithm outperforms all other tested registration methods, and the SyN presented as the next most effective method. However, the individual performance of registration algorithm across the various ROIs differs. For example, for the left ATR, the performance of SyN was slightly improved over DTI-TK.

### Evaluation method based on the MSE and RMSE of fibers

From Tables 3 and 4, smaller values of MSE and RMSE indicate a better registration as it shows the difference levels between each subject after registration and the template. As values for DTI-TK are the lowest, the DTI-TK registration algorithm was shown to be the most effective in this study with the SyN method ranking second.

**Table 4:** Evaluation results of registrations based on RMSE of fibers

	Genu	Splenium	L-ATR	R-ATR	L-CST	R-CST	L-IFO	R-IFO	Mean
Elastic	0.1683	0.2049	0.0830	0.0907	0.1257	0.1597	0.1124	0.0929	0.1297
Rigid	0.1739	0.2601	0.1393	0.1214	0.1967	0.2332	0.1297	0.1261	0.1726
Affine	0.2284	0.2584	0.1093	0.1112	0.1491	0.1910	0.1425	0.1087	0.1617
DTI-TK	0.0569	0.0963	0.0546	0.0546	0.0725	0.0800	0.0692	0.0597	0.0680
FSL	0.1645	0.2049	0.0876	0.0891	0.1266	0.1569	0.1149	0.0905	0.1294
SyN	0.0676	0.1173	0.0650	0.0678	0.0777	0.0872	0.0764	0.0755	0.0793

From Tables 3 and 4, smaller values of MSE and RMSE indicate a better registration as it shows the difference levels between each subject after registration and the template. As values for DTI-TK are the lowest, the DTI-TK registration algorithm was shown to be the most effective in this study with the SyN method ranking second.

### Evaluation method based on the FA profiles along the fiber tracts

**Fig. 5:**
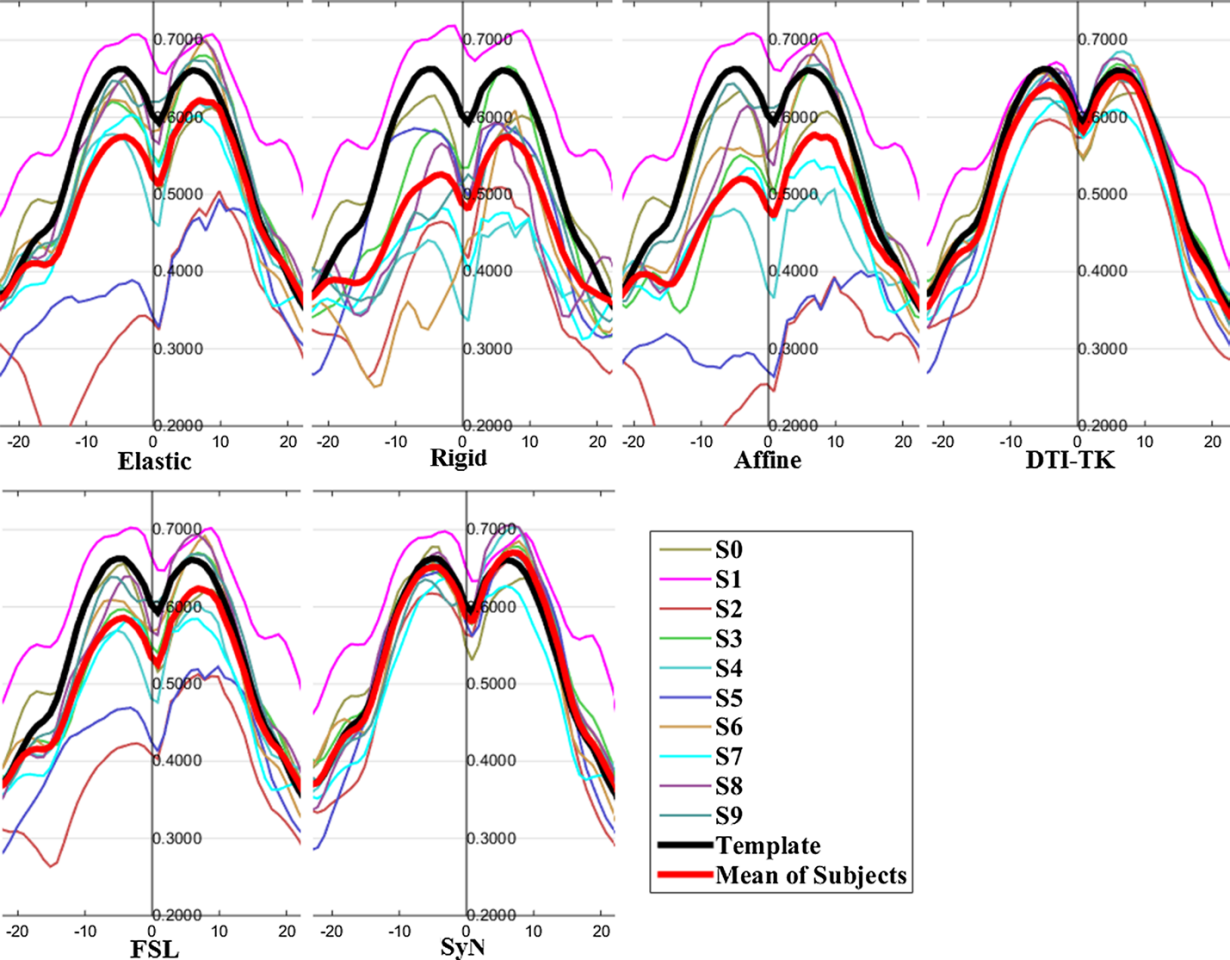
FA profiles of the Genu for the six registration methods

The FA profiles along the fiber tracts are shown in Fig. 5 through Fig. 12.

**Fig. 6:**
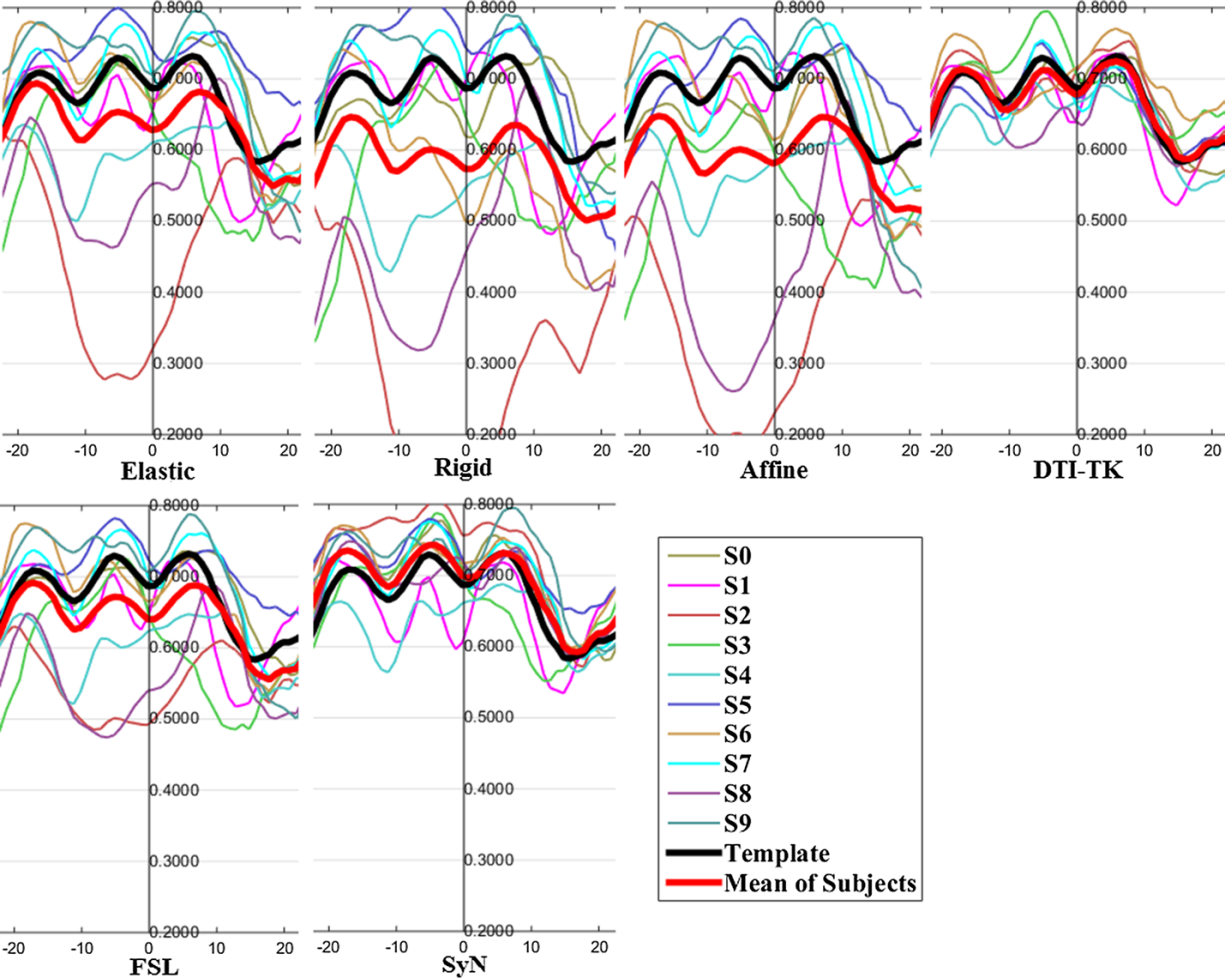
FA profiles of the Splenium for the six registration methods

**Fig. 7:**
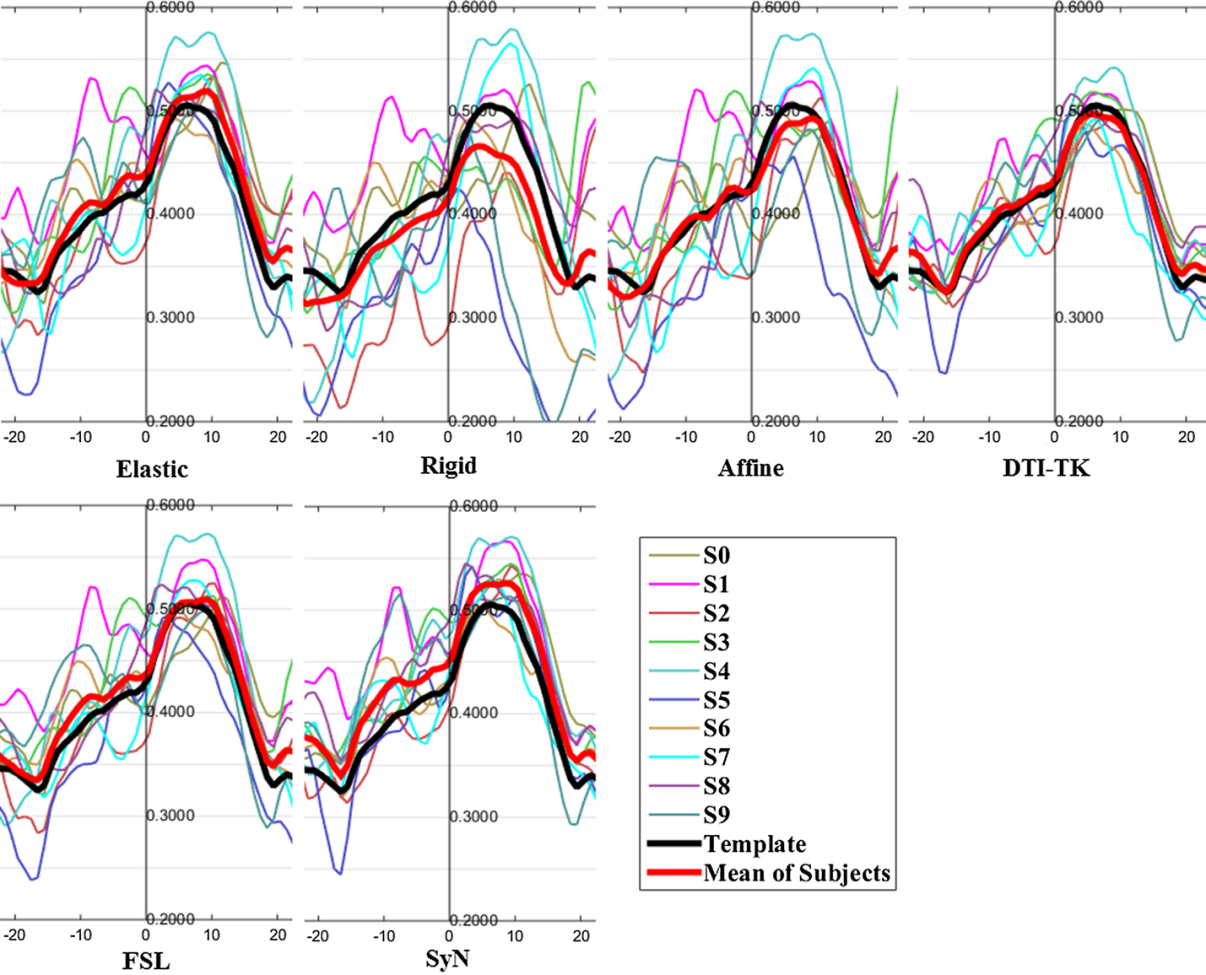
FA profiles of the *left* ATR for the six registration methods

**Fig. 8:**
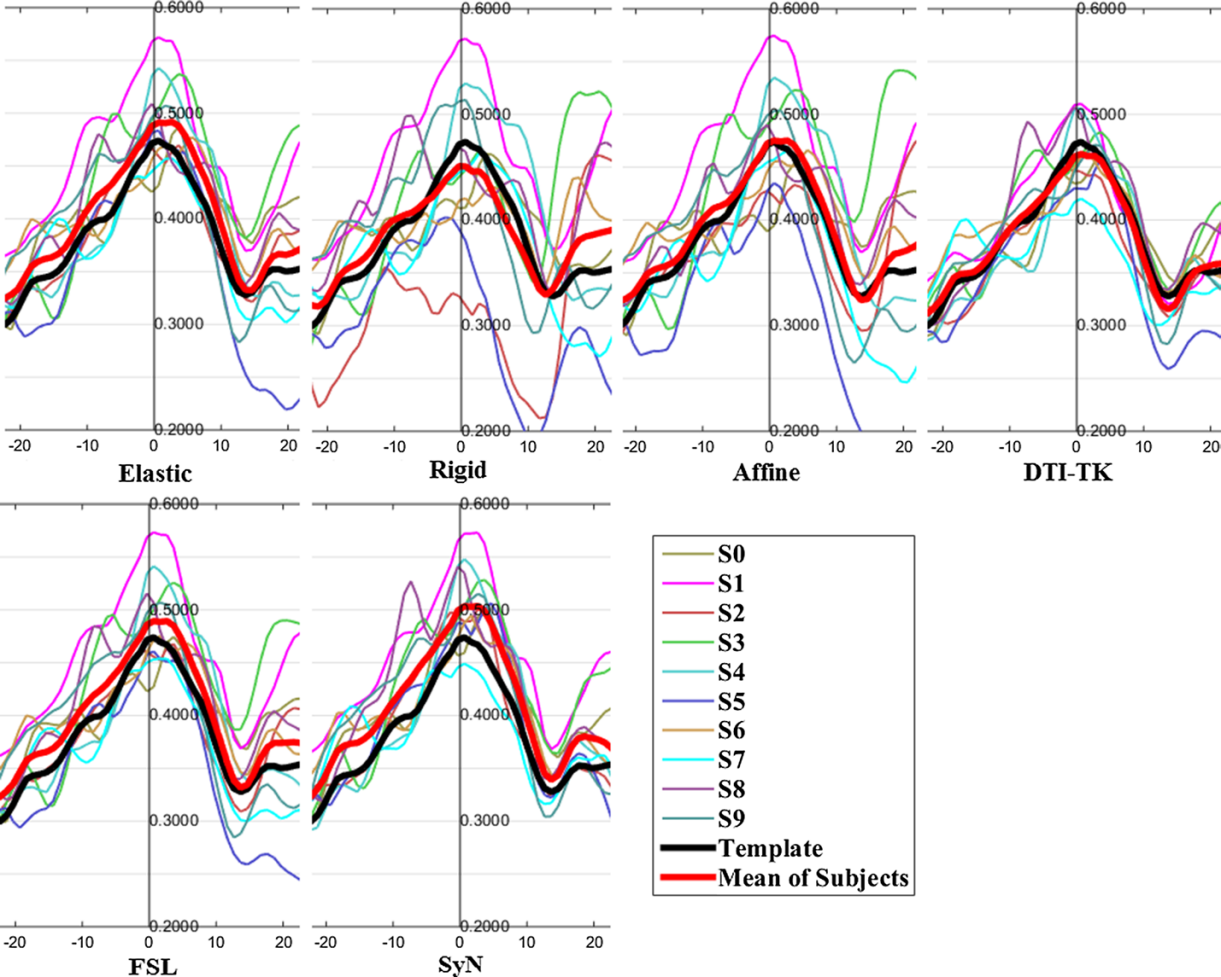
FA profiles of the *right* ATR for the six registration methods

**Fig. 9:**
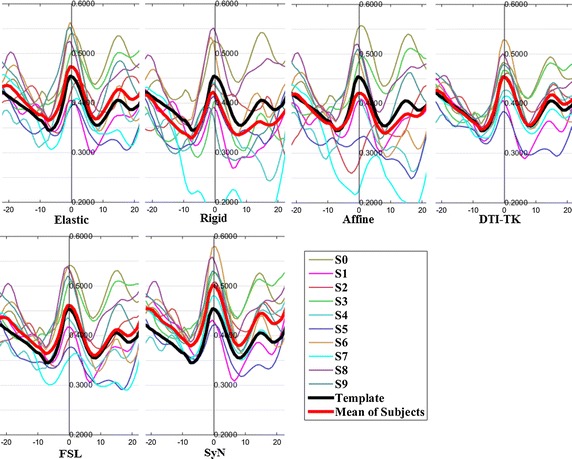
FA profiles of the *left* IFO for the six registration methods

**Fig. 10:**
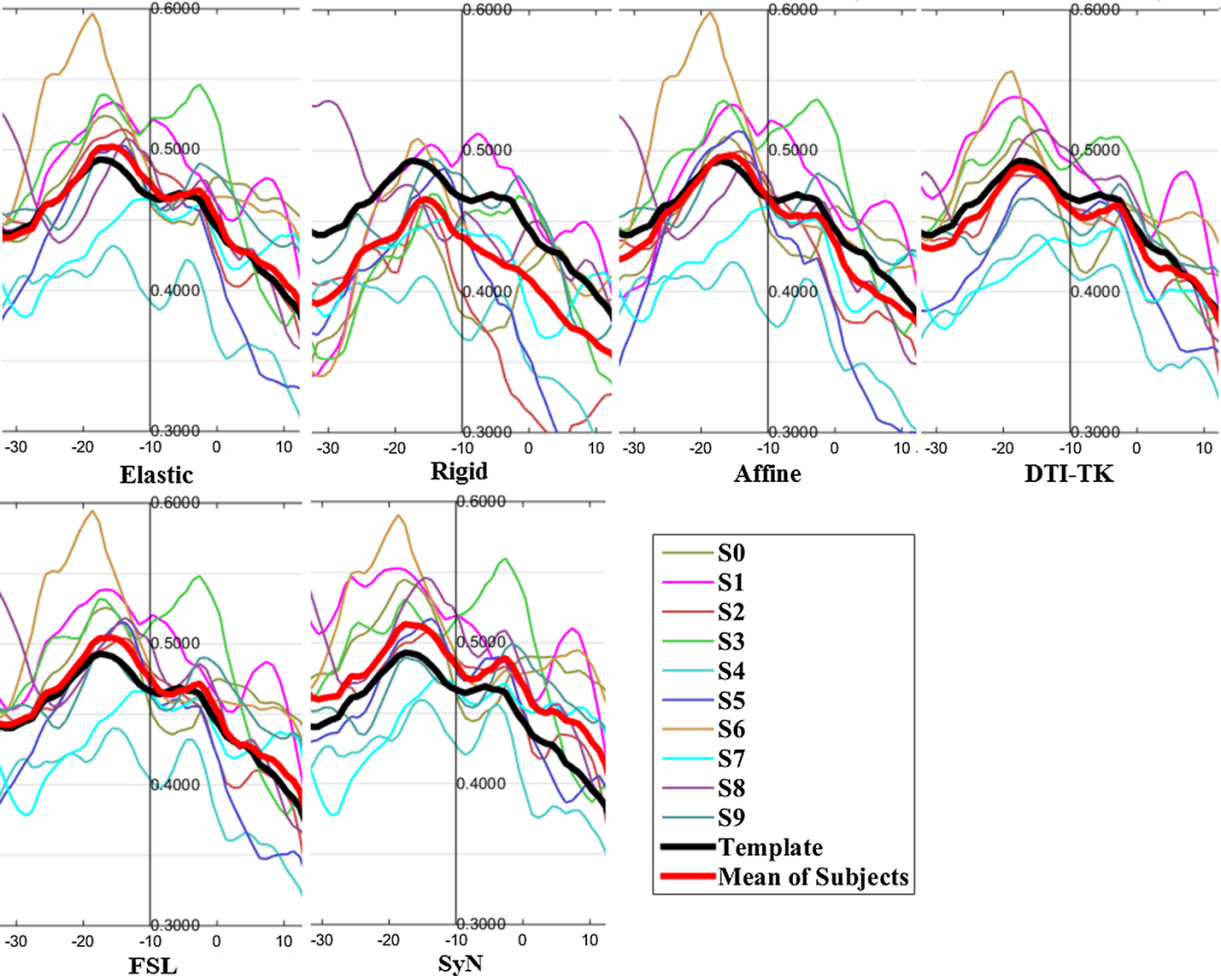
FA profiles of the *right* IFO for the six registration methods

**Fig. 11:**
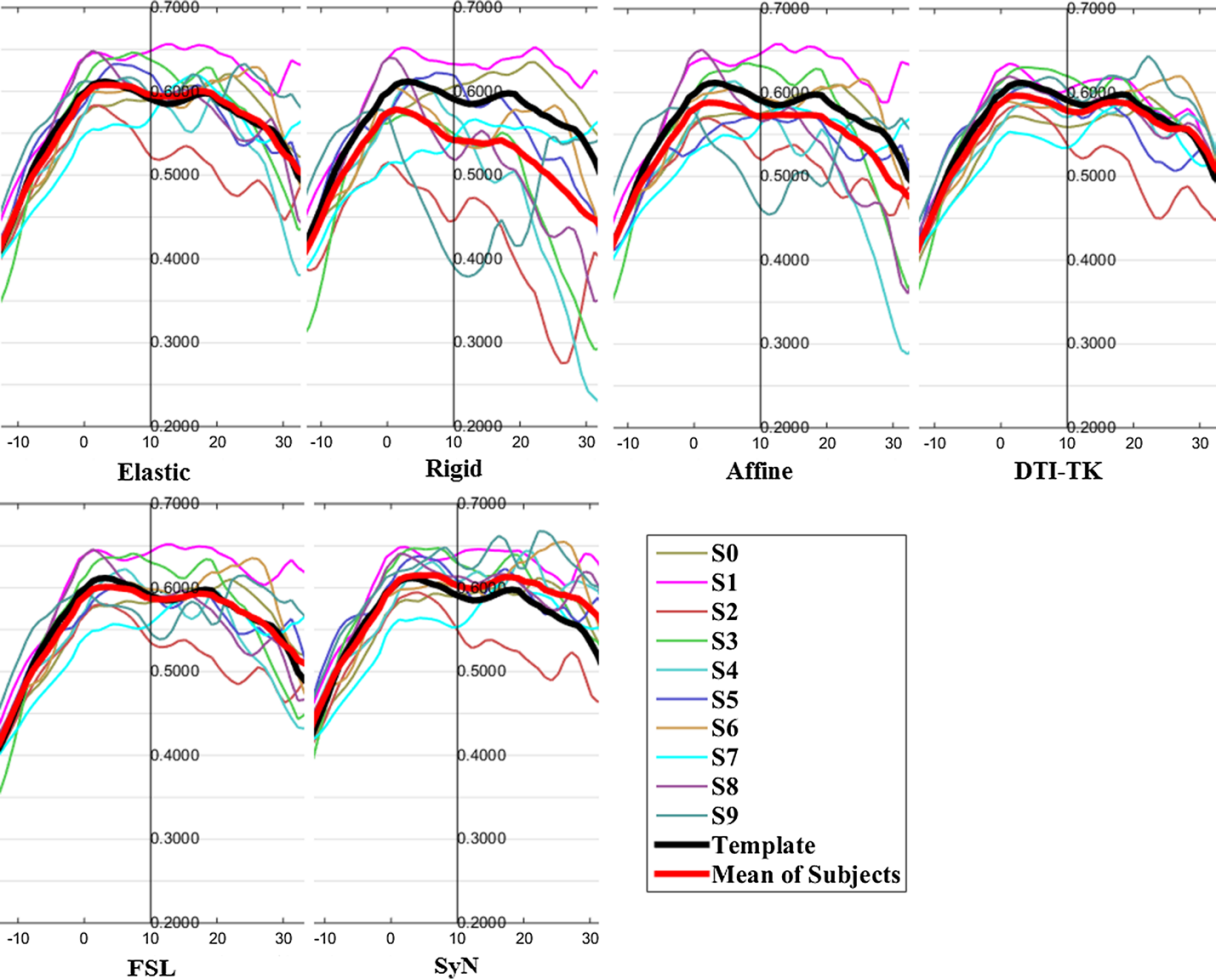
FA profiles of the *left* CST for the six registration methods

**Fig. 12:**
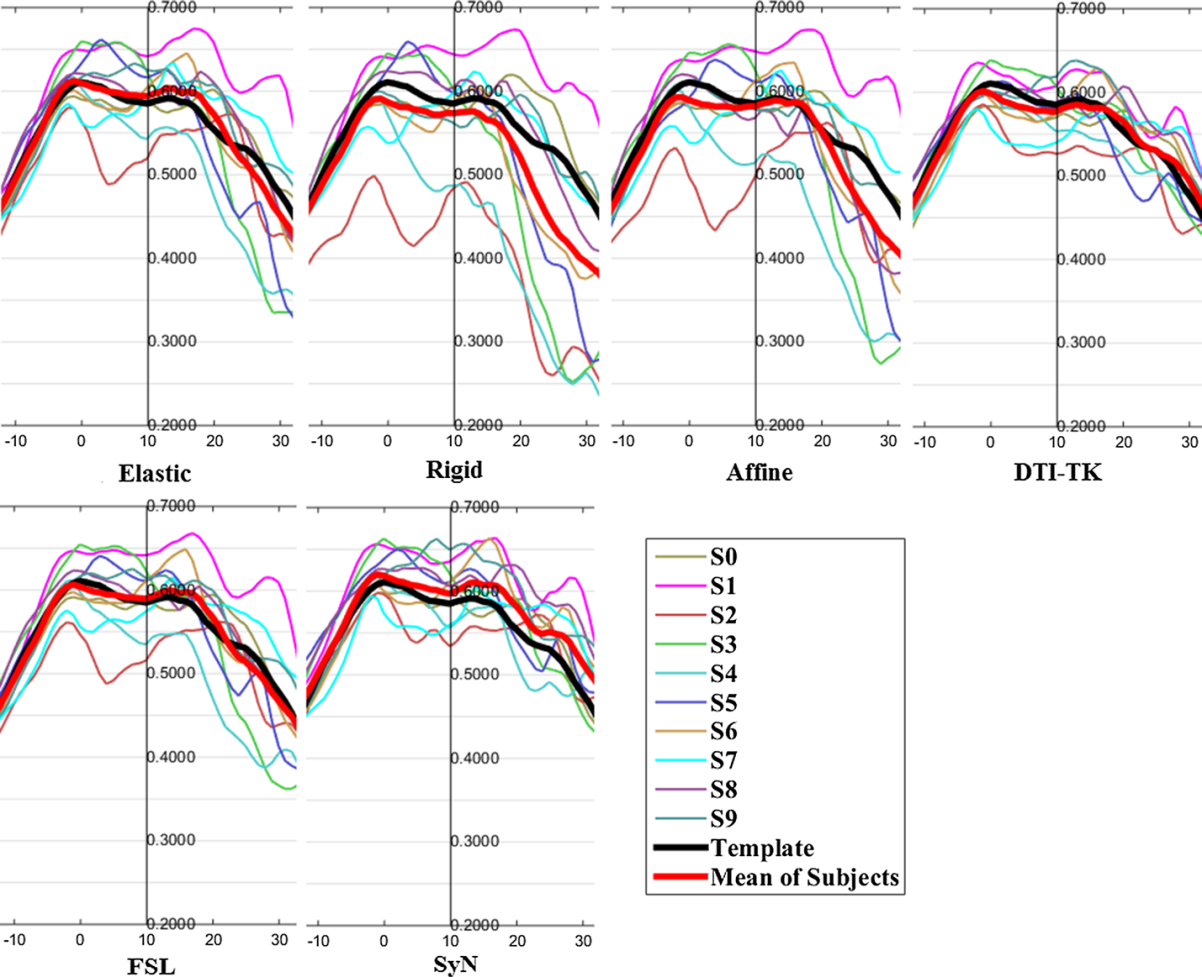
FA profiles of the *right* CST for the six registration methods

In these figures, the x-coordinate represents the arc length of the fiber bundles, and the y-coordinate is the value of FA. From the Figs. 5, 6, 7, 8, 9, 10, 11, 12, the FA profile characteristic curves of each subject obtained with DTI-TK are closest to the template (black color) and the mean of subjects (red color). The SyN and FSL algorithms ranked behind DTI-TK. However, as mentioned, the registration accuracy differs between the various ROIs and algorithms. For example, for the Genu structure, the Rigid algorithm performed better than the Affine, while for the Splenium structure, the Affine algorithm outperformed the Rigid algorithm.

Correlation coefficients between the FA profiles for each registered dataset and the template for all of the registration methods are shown in Table 5 for all subjects. Based on the correlation coefficients, no one algorithm outperformed the rest for all of the tracts. DTI-TK demonstrated the best results across eight ROIs, and performed well overall. However, it is difficult to identify one algorithm as the best based on the normative tract profile correlation evaluation across all fiber tracts.

**Table 5:** Correlation coefficients between FA profiles of various fiber tracts on registered subjects and the template for the six registration algorithms

	Elastic	Rigid	Affine	DTI-TK	FSL	SyN	Best
*Genu*							
MEAN	0.4317	0.3890	0.4101	0.4331	0.4337	0.4572	DTI-TK
STDEV	0.0974	0.0963	0.0794	0.1324	0.1001	0.1220	
*p* value	0.8721	0.8628	0.7364	0.9854	0.9464	0.9784	
Rank	4	5	6	1	3	2	
*Splenium*							
MEAN	0.4524	0.4239	0.4312	0.4554	0.4624	0.4935	DTI-TK
STDEV	0.1285	0.1118	0.1140	0.1527	0.1260	0.1393	
*p* value	0.8773	0.7255	0.7794	0.9726	0.9137	0.9488	
Rank	4	6	5	1	3	2	
*Left ATR*							
MEAN	0.3945	0.3695	0.3876	0.3818	0.3966	0.4056	DTI-TK
STDEV	0.0602	0.0552	0.0559	0.0663	0.0595	0.0654	
*p* value	0.7731	0.5686	0.6687	0.8882	0.8170	0.8785	
Rank	4	6	5	1	3	2	
*Right ATR*							
MEAN	0.3876	0.3679	0.3793	0.3840	0.3930	0.4068	DTI-TK
STDEV	0.0666	0.0609	0.0586	0.0783	0.0681	0.0806	
*p* value	0.7928	0.6317	0.6886	0.9120	0.8535	0.9091	
Rank	4	6	5	1	3	2	
*Left CST*							
MEAN	0.4374	0.4071	0.4257	0.4282	0.4435	0.4613	DTI-TK
STDEV	0.1056	0.0965	0.1016	0.1070	0.0982	0.0968	
*p* value	0.9002	0.8189	0.8700	0.9507	0.9259	0.9376	
Rank	4	6	5	1	3	2	
*Right CST*							
MEAN	0.4634	0.4256	0.4490	0.4633	0.4723	0.4956	DTI-TK
STDEV	0.1105	0.1113	0.1088	0.1154	0.1067	0.1072	
*p* value	0.8509	0.8121	0.8249	0.8249	0.9095	0.9239	
Rank	4	6	5	1	3	2	
*Left IFO*							
MEAN	0.3890	0.3629	0.3691	0.3843	0.3918	0.4145	DTI-TK
STDEV	0.0609	0.0618	0.0547	0.0638	0.0580	0.0628	
*p* value	0.6879	0.6470	0.5858	0.8446	0.7382	0.8117	
Rank	4	5	6	1	3	2	
*Right IFO*							
MEAN	0.4110	0.3695	0.3988	0.3958	0.4148	0.4302	DTI-TK
STDEV	0.0549	0.0563	0.0528	0.0687	0.0536	0.0566	
*p* value	0.7669	0.6525	0.6994	0.9072	0.7918	0.8124	
Rank	4	6	5	1	3	2	

Additionally, based on the correlation coefficients, we considered the correlation values of 0.85 [2] as the threshold when fiber tracts were mapped to the template. Correlation coefficients below the threshold were marked as a failure. Table 6 shows the number of failures when eight DTI fiber bundles were mapped to the template for the ten subjects. The DTI-TK algorithm resulted in the minimum number of failures and can be considered the best algorithm based on this criterion.

**Table 6:** Number of failures in mapping the subject fiber tracts to the template with a correlation value greater than 0.85 for the six registration algorithms

	Elastic	Rigid	Affine	DTI-TK	FSL	SyN
Genu	2	3	3	0	1	0
Splenium	2	4	5	0	1	0
Left ATR	9	10	10	2	7	2
Right ATR	9	9	9	0	4	1
Left CST	1	5	2	0	1	1
Right CST	1	4	2	0	1	0
Left IFO	9	8	10	4	9	5
Right IFO	7	9	8	2	5	5

### Evaluation method based on intersection angles between fiber bundles

In Table 7, average cosine values of the intersection angles between each subject and template tracts are shown.

**Table 7:** Average cosine values of intersection angles between each subject and the template tracts

	Genu	Splenium	L-ATR	R-ATR	L-CST	R-CST	L-IFO	R-IFO	Mean
Ealstic	0.8601	0.7746	0.8330	0.8428	0.8349	0.8134	0.8484	0.8423	0.8312
Rigid	0.7746	0.7040	0.7889	0.7908	0.7793	0.7606	0.8078	0.7657	0.7715
Affine	0.8276	0.7434	0.8090	0.8209	0.8097	0.7846	0.8178	0.8163	0.8037
DTI-TK	0.9164	0.8491	0.8849	0.8923	0.8956	0.8762	0.9132	0.9010	0.8911
FSL	0.8840	0.8008	0.8753	0.8785	0.8764	0.8152	0.8966	0.8848	0.8220
SyN	0.8850	0.8062	0.8607	0.8576	0.8509	0.8376	0.8786	0.8693	0.8557

From the average $\cos \alpha$ values of six registration algorithms in Table 7 the value of $\cos \alpha$ in DTI-TK is the largest, which means the angle is the smallest. The cosine value of SyN is larger than the other registration algorithms except DTI-TK. In conclusion, the DTI-TK registration algorithm performed the best, and the SyN ranked second as observed with other evaluation methods.

### Evaluation method based on spatial similarity between fiber tracts

**Fig. 13:**
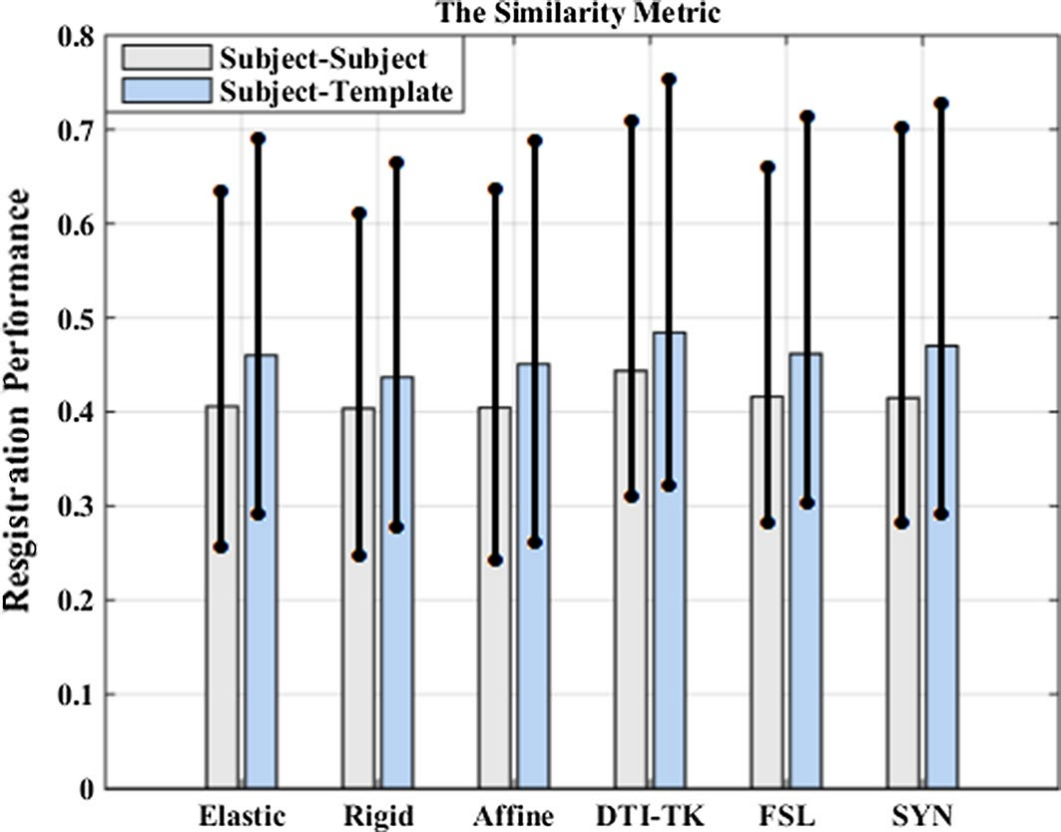
The average similarity metric of six different registration algorithms

Figure 13 shows the spatial similarity metric of fibers between each subject and subject pairs, along with the similarity metric of fibers between each subject and template pairs. From Fig. 13, DTI-TK had the largest average similarity across the six registration algorithms, and the average similarity from the SyN algorithm is larger than remaining registration algorithms. Similar to previous evaluations, the DTI-TK registration algorithm performed the best, and the SyN algorithm ranked second.

## Discussion

In this paper, we used deterministic tractography for fiber tracking and evaluated six registration methods with the distance between fibers of subjects and the template, the MSE and RMSE, the average FA profiles, and angles between fibers of subjects and the template. From Table 2, the average distance of DTI-TK was smallest, which implied DTI-TK is the best, but it was not the smallest across all ROIs. For example, in the Genu ROI, the distance determined with SyN was smaller than that with DTI-TK. From Tables 3 and 4, results of MSE and RMSE show that the average values for DTI-TK were the smallest. However across the various ROIs, no single method performed the best for all ROIs. From Figs. 5, 6, 7, 8, 9, 10, 11, 12 and Tables 5 and 6, the six registration algorithms were easily ranked and the results are basically the same. Only the results for the Affine and Rigid algorithms differed between a few ROIs. The *p* values in Table 5 show that correlation coefficients obtained with DTI-TK are the highest. Further, in Table 6, DTI-TK had the minimum number of failures using the selected threshold and can be considered as the best algorithm based on that criterion. According to the average FA profile evaluation, DTI-TK seemed to show the best registration performance. Based on the angles between fibers of subjects and the template (Table 7) evaluation, similar to the distances between fibers of subjects and the template evaluation, DTI-TK again showed the best registration performance because the value of $\cos \alpha$ in DTI-TK is the largest, which means the intersection angle is the smallest. However, the registration algorithms did not always perform the best for all ROIs in a single subject, and may be due to the fact that since subjects and the template were chosen at random for this study, the differences in registration performance across the six registration algorithms as observed on full tract evaluation. The performance of DTI-TK in correctly mapping the eight fiber tracts for all subjects can be attributed to the fact that the algorithm exploits the whole tensor orientation information for the registration compared to the scalar FA values.

We also used probabilistic tractography for fiber tracking and evaluated the six registration methods with a spatial correlation similarity metric. Spatial correlation as a similarity measurement provides a precise and reproducible evaluation of registration quality when using the appropriate framework [12] which is based on multiple tracts identified with probabilistic tractography. From Fig. 13, the spatial similarity metric of fibers between subjects shows DTI-TK was the best. To avoid occasional bias observed with the comparison of different subjects, we also calculated the spatial similarity metric of fibers between each subject and the template, which again indicated that DTI-TK outperformed the rest of the algorithms. It should be mentioned that the spatial similarity values were the average of all the ROIs across all subjects.

Registration performance measurements based on deterministic tractography of different ROIs are not always same as those based on probabilistic tractography. Again from Fig. 13, the spatial similarity metric calculated on pairs of subjects and individual subjects differed, similarly as in the calculation for pairs of subjects and template. As increasing the subjects would reduce the random error, future work would include a larger study cohort, and a template based on all of the subjects. We would also like to expand the ROIs chosen for analysis.

At the moment, evaluation methods based on deterministic tractography are gradually maturing; however, methods based on probabilistic tractography are still in the primary stage of development [12]. When tracking the fibers, probabilistic tractography still requires much more calculation time than deterministic tractography [2, 5, 12]. Reduction of the tracking time in probabilistic tractography and development of new evaluation methods based on probabilistic tractography are areas of ongoing research.

## Conclusions

In this paper, six open source registration algorithms were applied with randomly chosen subjects from IXI dataset and evaluated based on fiber tracts obtained through deterministic and probabilistic tractography. Results indicated that the DTI-TK and SyN registration algorithms outperformed the other registration algorithms overall. In conclusion, DTI-TK qualifies as the best registration algorithm, and SyN ranks just behind DTI-TK for the evaluation techniques studied. It should be noted that results from criteria based on deterministic tractography are not the same as those based on probabilistic tractography. For example, the Affine registration algorithm is generally considered as the worst based on deterministic tractography while the Rigid registration algorithm is the worst based on probabilistic tractography.

## Abbreviations

DT-MRI: diffusion tensor magnetic resonance imaging
ROIs: regions of interest
FA: fractional anisotropy
MSE: mean squared error
RMSE: residual MSE
DTI-TK: diffusion tensor imaging toolkit
BET: brain extraction tool
FSL: FMRIB software library
DWIs: diffusion-weighted images
ANTs: advanced normalization tools
PPD: preservation of principal directions
FACT: fiber assessment by continuous tracking
ATR: anterior thalamic radiations
IFO: inferior fronto-occipital fasciculi
CST: corticospinal/corticobulbar tracts
